# Recurrent Nonbacterial Thrombotic Endocarditis and Primary Antiphospholipid Antibody Syndrome

**DOI:** 10.7759/cureus.36275

**Published:** 2023-03-17

**Authors:** Amit K Mandal, Randeep S Heer, Mario Petrou, Constantinos G Missouris

**Affiliations:** 1 Cardiology/Internal Medicine, Wexham Park Hospital, Frimley Health NHS Foundation Trust, Slough, GBR; 2 Cardiac Surgery, The Royal Brompton and Harefield NHS Foundation Trust, London, GBR; 3 Cardiology, University of Nicosia Medical School, Nicosia, CYP

**Keywords:** nonbacterial thrombotic endocarditis, valve repair, surgical replacement of valve, non-vitamin k oral anticoagulant, marantic endocarditis, recurrent endocarditis, primary antiphospholipid antibody syndrome, nonbacterial thrombotic endocarditis (ntbe)

## Abstract

In 2015, a 37-year-old man was referred for evaluation of hypertension and was found to have a mobile structure on the posterior mitral valve leaflet on echocardiography. Laboratory investigations yielded a diagnosis of primary antiphospholipid antibody syndrome (APLS). He underwent excision of the lesion and mitral valve repair. Histology confirmed the diagnosis of nonbacterial thrombotic endocarditis (NBTE). The patient was anticoagulated with warfarin up until 2018, which was substituted for rivaroxaban because of an erratic international normalised ratio. Serial echocardiography up to 2020 was unremarkable. In 2021, he presented with breathlessness and peripheral oedema. Echocardiography demonstrated large vegetation on both mitral valve leaflets. At the operation, vegetations were also evident on the left and noncoronary cusps of the aortic valve and he underwent mechanical aortic and mitral valve replacement. Histology confirmed NBTE. The case is unusual and highlights recurrent NBTE requiring re-do valve surgery.

## Introduction

Antiphospholipid antibody syndrome (APLS) is an autoimmune condition characterized by recurrent arterial and venous thrombosis and miscarriage. It can be classified as primary or secondary depending on the presence or absence of related rheumatic or autoimmune diseases such as systemic lupus erythematosus. In this case, we highlight the rare association of APLS and nonbacterial thrombotic endocarditis (NBTE), also known as Libman-Sacks and marantic endocarditis [1,2]. After surgical resection and preservation of the native valve, it remains as a substrate for the recurrence of NBTE. Albeit with little available evidence, valve replacement may therefore be superior. Also, the necessity of anticoagulation with vitamin K antagonists, over direct oral anticoagulants, and younger age of patients with APLS may favour mechanical over bioprosthetic valve replacement.

## Case presentation

**Figure 1:**
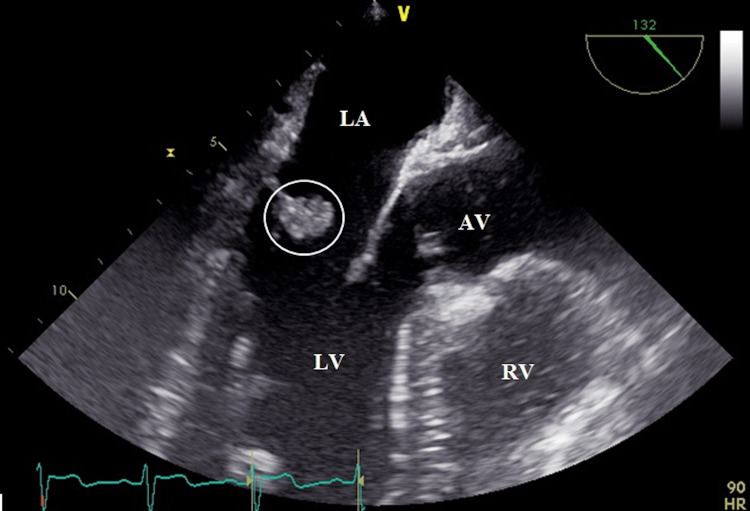
Transoesophageal echocardiography demonstrating an echodensity on the atrial aspect of the posterior mitral valve leaflet AV: aortic valve; LA: left atrium; LV: left ventricle; RV: right ventricle.

In 2015, an asymptomatic 37-year-old man with hypertension was referred for echocardiography to evaluate cardiac structure and function and assess left ventricular mass. There was no other relevant past medical history. Examination revealed a grade 3/6 pansystolic murmur at the left sternal edge, normal apex, no signs of heart failure, and no peripheral stigmata of endocarditis. Transthoracic echocardiography (TTE) and transoesophageal echocardiography (TOE) demonstrated a structure suggestive of vegetation on the posterior mitral valve leaflet with moderate regurgitation (Figure 1).

Inflammatory markers were normal and without features of bacterial infection. Repeated cultures were negative. Serological tests for *Coxiella burnetii* were negative. The extended autoimmune and rheumatic screen was negative bar the presence of lupus anticoagulant and strongly positive anti-cardiolipin antibodies. Pan-computed tomography did not reveal cancer. This allowed a diagnosis of primary (idiopathic) APLS, which had remained clinically dormant [1].

The patient received empirical intravenous antibiotics to cover left side native valve endocarditis and was anticoagulated with warfarin (target international normalised ratio (INR): 2.0-3.0). For definitive management and to achieve a diagnosis, the patient underwent surgical excision and mitral valve repair. Histological features were typical of NBTE consisting of fibrin strands, inflammatory infiltrates, and erythrocytes. The patient made an uneventful recovery and continued anticoagulation with warfarin up until 2018 when this was substituted for rivaroxaban because of erratic INR. Serial TTE up to 2020 demonstrated a stable appearance of all cardiac valves and the patient did not experience thromboembolic or haemorrhagic events.

**Figure 2:**
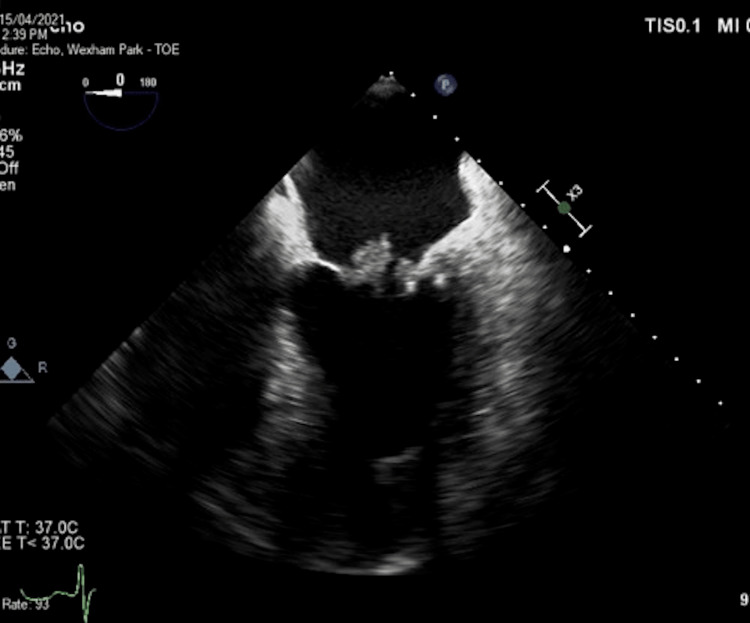
Transoesophageal echocardiography illustrating vegetations on both mitral valve leaflets

In 2021, the patient re-presented with a short history of breathlessness and peripheral oedema. He had remained compliant with rivaroxaban. There were no peripheral stigmata of endocarditis. TOE demonstrated large fleshy vegetations on both mitral valve leaflets with severe regurgitation (Figure 2). Laboratory investigations were unremarkable without bacteraemia. Again, the patient was treated empirically with intravenous antibiotics and transferred for surgical intervention. At operation, vegetation was also found on the left and noncoronary cusps of the aortic valve with moderate regurgitation (unfortunately, we do not have the intraoperative TOE images). Considering his young age, the patient underwent uncomplicated mechanical aortic and mitral valve replacement. Histology of the valvular tissue confirmed NBTE. The patient was prescribed lifelong warfarin.

## Discussion

NBTE, or ‘marantic endocarditis’, is characterised by sterile vegetations involving the endocardium and cardiac valves. It is associated with hypercoagulable states, especially in the context of adenocarcinomas, and autoimmune diseases such as systemic lupus erythematosus and APLS. It carries a high risk of systemic embolisation [1].

APLS is typically diagnosed between the ages of 30 and 40 years, and it affects three to five times as many women as men. Primary APLS, where there is no evidence of autoimmune disease, as in our patient, is an exceedingly rare association of NBTE. A major difference between primary and secondary APLS is the pattern of affected valves. In primary APLS, NBTE affects aortic and mitral valves equally, whereas in secondary APLS, there is a predominance (75%) for the mitral valve [2].

The management of NBTE relies on identifying and treating the underlying pathology accordingly. Because of its rarity, there is no consensus or strong evidence-based guidance. Anticoagulation and surgical excision (which expedites the diagnosis) theoretically improve cardiovascular outcomes. Vitamin K antagonists are presently the mainstay treatment for NBTE in patients with autoimmune disease and APLS. For patients with cancer-related coagulopathy, the available evidence supports the use of intravenous or subcutaneous heparins. Emerging data suggest direct oral anticoagulants, particularly rivaroxaban, have a limited role [3,4].

There are longitudinal series of patients with idiopathic APLS and NBTE where progressive valve destruction is described despite continuing anticoagulation. The risk of progression appears to be associated with higher titres of anti-cardiolipin antibodies and positive lupus anticoagulants [5]. Our case is of interest as it is one of a few describing recurrence of NBTE after surgical resection requiring a re-do sternotomy and valve replacement [6]. Notably, the resurgent disease process was florid with unusually abundant and corpulent vegetation. Patients with APLS should have regular surveillance echocardiography as should all patients with NBTE when the causal pathology has a reasonable prognosis. Furthermore, these patients should receive lifelong anticoagulation with vitamin K antagonists, even in the absence of a mechanical valve prosthesis.

## Conclusions

Although anticoagulation may prevent thromboembolic phenomena, it is unlikely to have any impact on the progression or prevention of valve lesions. Given that the initial surgery in our patient was a vegetectomy, the native mitral and aortic valves remained as a substrate for recurrence, and valve replacement might be superior to surgical preservation of the affected valve in the prevention of relapse. The necessity of anticoagulation with warfarin, alongside the relatively younger age of patients with primary and secondary APLS, favours mechanical over bio-prosthetic valve replacement; the caveat being that, even with therapeutic INR, any thrombotic complication can easily obstruct the hinge mechanism.
